# Gender Differences in the Consequences of Divorce: A Study of Multiple Outcomes

**DOI:** 10.1007/s13524-018-0667-6

**Published:** 2018-04-13

**Authors:** Thomas Leopold

**Affiliations:** University of Amsterdam, Nieuwe Achtergracht 166, 1018 WV Amsterdam, The Netherlands

**Keywords:** Divorce consequences, Gender inequalities, Adult outcomes, Fixed-effects models, Germany

## Abstract

In this study, I examined gender differences in the consequences of divorce by tracing annual change in 20 outcome measures covering four domains: economic, housing and domestic, health and well-being, and social. I used data from the German Socio-Economic Panel Study (SOEP) and fixed-effects panel regression models on a sample of *N* = 18,030 individuals initially observed in a marital union, *N* = 1,220 of whom divorced across the observation period (1984–2015). Three main findings emerged from the analysis. First, men were more vulnerable to short-term consequences of divorce for subjective measures of well-being, but postdivorce adaptation alleviated gender differences in these outcomes. Second, a medium-term view on multiple outcomes showed more similarity than differences between women and men. The medium-term consequences of divorce were similar in terms of subjective economic well-being; mental health, physical health, and psychological well-being; residential moves, homeownership, and satisfaction with housework; and chances of repartnering, social integration with friends and relatives, and feelings of loneliness. Third, the key domain in which large and persistent gender differences emerged were women’s disproportionate losses in household income and associated increases in their risk of poverty and single parenting. Taken together, these findings suggest that men’s disproportionate strain of divorce is transient, whereas women’s is chronic.

**Electronic supplementary material:**

The online version of this article (10.1007/s13524-018-0667-6) contains supplementary material, which is available to authorized users.

## Introduction

Who suffers more from divorce: men or women? Debates about gender differences in the consequences of divorce as well as policies aimed at alleviating these differences often center on women’s vulnerability (Amato 2000; Diedrick 1991). After divorce, women experience disproportionate declines in household income (de Vaus et al. 2015; Smock 1994) and standard of living (Bianchi et al. 1999; Peterson 1996) as well as sharp increases in the risk of poverty (Smock and Manning 1999). Women may also face a higher risk of losing homeownership and “falling down the housing ladder” (Dewilde 2008). Women’s lower chances of repartnering (Wu and Schimmele 2005) and responsibilities as a single parent may further impede their path to economic recovery.

This view of women bearing the highest burden of divorce and requiring more public and private support than their ex-partners is partly based on solid evidence. Yet, the seemingly clear picture gets clouded when put into a larger context of divorce outcomes. Divorce effects, and gender differences therein, extend into various spheres, including changes in economic status, health and well-being, domestic arrangements, and social relationships. In these domains, several studies have reported that men were more vulnerable to the adverse effects of divorce, including larger health declines and lower subjective well-being after separation (Shor et al. 2012; Stack and Eshleman 1998), higher risk of adopting bad health habits (Umberson 1992), elevated mortality (Berntsen and Kravdal 2012; Sbarra et al. 2011), disproportionate declines in satisfaction with family life (Leopold and Kalmijn 2016), higher dissatisfaction with custodial arrangements (Bauserman 2012; Sheets and Braver 1996), and greater feelings of loneliness and social isolation (Dykstra and Fokkema 2007). Although the evidence is not consistent about all these effects, it suggests that an assessment of gender differences in the consequences of divorce should look at multiple outcomes.

Yet, extant studies of divorce effects on adults have predominantly focused on only one outcome or on a set of outcomes within one domain—most commonly, economic well-being or health. Studies that cut across two or more domains are rare. This gap of research precludes a broader view of gender differences in the multiple consequences of divorce. To obtain a fuller picture, an analyst has to piece together evidence from a large literature that varies in terms of sampling frames, longitudinal scope, methods of analysis, and the societal and historical context from which the data were drawn. As a result of this heterogeneity, the empirical basis for broader conclusions about gender differences in the consequences of divorce remains limited.

To address this limitation, with the present study, I aimed to offer a comprehensive view of gender differences in the consequences of divorce by tracing annual change in multiple measures covering four outcome domains: economic, housing and domestic, health and well-being, and social. Although these four domains are interrelated and partly overlapping, this classification is useful as an organizing scheme for relevant outcomes and related findings.

I analyzed data from 32 waves (1984 until 2015) of the German Socio-Economic Panel Study (SOEP), one of the world’s largest and longest-running household panel studies. An important benefit of these data is the large array of subjective and objective outcome measures combined with an extensive window of observation, allowing me to assess short-term and medium-term consequences of divorce as well as gender differences therein. My sample included 18,030 individuals initially observed in a marital union, 1,220 of whom divorced across the observation period (1984–2015). The analysis was based on fixed-effects models for within-person change occurring up to 5 years before and after divorce.

## Background

### Economic Outcomes of Divorce

Numerous studies have shown that the economic costs of divorce fall more heavily on women. After separation, women experience a sharper decline in household income and a greater poverty risk (Smock 1994; Smock and Manning 1999). Their former husbands, in contrast, may even improve their standard of living in postdivorce years. Peterson (1996) quantified the resulting gender gap for the United States, estimating a 27 % decline among women and a 10 % increase among men in their standard of living. Other U.S. estimates for women’s drops in economic well-being are even larger (Bianchi et al. 1999). Similar results were found for the German context of the present study: Andress and Bröckel (2007) found that women’s household incomes 1 year after divorce amounted to only two-thirds of those of their former husbands.

Explanations for these gender inequalities highlight four risk factors for women (Bröckel and Andress 2015; Holden and Smock 1991): (1) higher economic need and restricted earning capacities in the presence of children; (2) insufficient child maintenance; (3) disproportionate loss of income, which is often not fully compensated by spousal maintenance; and (4) human capital deficits resulting from gender specialization in the division of labor during marriage.

Although the evidence shows that divorce hits women harder in terms of economic outcomes, two qualifications apply to this conclusion. First, few studies have examined whether women’s economic strain is chronic. A recent comparative study indicated that in Germany, short-term effects are larger than medium-term effects: women’s incomes recovered in the years after divorce (de Vaus et al. 2015).

Second, results may look different for subjective measures of economic well-being. Theoretical models of the divorce process—notably, the crisis model and the chronic strain model (Johnson and Wu 2002; McLanahan and Sandefur 1994)—have stressed the importance of how individuals subjectively experience changes in their economic status. Knowledge about gender differences in subjective measures of economic well-being, however, remains scarce. An early study (Keith 1985) concluded that women were more satisfied with their financial status after separation than men. Findings for the German context have shown that women’s satisfaction with household income reached men’s levels shortly after separation (Andress and Bröckel 2007; Leopold and Kalmijn 2016). These results suggest that research should consider both objective and subjective measures to understand gender differences in postdivorce economic well-being.

### Housing and Domestic Outcomes of Divorce

Housing and domestic outcomes figure prominently among the stressors associated with the divorce process. One line of research in this area has asked whether men or women are more likely to move out after separation. According to rational choice models, the question of who moves out is answered on the basis of each partner’s resources and costs associated with staying and moving, including direct costs of moving but also costs in terms of disrupting ties to family, friends, and the workplace (Mulder and Wagner 2010). Although some of the relevant costs and resources are gendered, these differences seem to balance out on a larger scale. For example, Dutch women were more likely to leave the shared household in the absence of children, but the reverse was true in the presence of children (Feijten and Mulder 2010; Mulder and Wagner 2012). On average, male and female partners in the Netherlands were almost equally likely to move out after separation. Evidence for other countries, such as the UK and Sweden, also did not point to major gender differences in the risk of moving out after separation (Feijten and Mulder 2010; Mulder and Malmberg 2011).

A second line of research has looked at changes in homeownership. Given that divorce constitutes a major life course risk of losing homeownership, a number of housing studies have examined gender inequality in this risk. A guiding idea behind these studies is that women are, on average, more dependent on their partners and therefore at a higher risk of losses in terms of quality and security of housing after divorce. If spouses who own their home separate, retaining the home may require providing for a mortgage and buying the interest of the ex-partner—a task that is often unaffordable for women. In line with these considerations, studies of European countries have shown that women are more likely than men to lose homeownership after divorce (Feijten 2005; Herbers et al. 2014). For the German setting of the present study, high levels of gender specialization and low levels of women’s labor force participation may contribute to these differences. However, the German welfare state provides for those with financial need, potentially facilitating women’s economic recovery and alleviating the negative association between divorce and homeownership (Dewilde and Stier 2014).

A third line of research on housing and domestic outcomes of divorce has examined implications for the performance of housework and the gendered division of household labor. Although studies have focused more on the reverse direction of this relationship (i.e., how gender roles in the home affect the risk of divorce), some have addressed the effects of divorce on the performance of housework. Two-wave panel studies have shown that men substantially increased their time spent on routine housework after separation, whereas women moderately reduced their housework hours (Baxter et al. 2008; Gupta 1999). A multiwave panel study indicated that these changes may be permanent (Hewitt et al. 2013). To the extent that routine housework can be considered an onerous activity, these findings suggest that women experience a moderate relief in this domain, whereas men’s domestic well-being is more strongly, and more negatively, affected. The latter might apply particularly to men who endorse traditional gender role attitudes. Among those men, greater involvement in female-typed activities might exacerbate divorce-related stress by adding dissonance to their gender identity (West and Zimmerman 1987). To gain more insight into these issues, it is useful to complement objective measures of hours spent on routine housework by subjective measures, such as satisfaction with performing these tasks.

### Health and Well-being Outcomes of Divorce

Early studies that compared divorced men with divorced women concluded that postdivorce adaptation in health and well-being favors women (Stack and Eshleman, 1998; Wallerstein 1986). One explanation for these differences relates to gendered health benefits of marriage: because men experience greater health gains from marriage, divorce puts them at a higher risk of health declines and mortality. In line with this idea, more recent research has indicated that life satisfaction was lower among divorced men (Andress and Bröckel 2007) and that mortality following divorce increased only among men (Berntsen and Kravdal 2012; Shor et al. 2012).

A second explanation highlights behavioral differences in the predivorce period. Women are more aware of marital problems and make greater investments in holding a marriage together (Baruch et al. 1983). At the same time, women are more likely to initiate divorce after they accept that their efforts are hopeless (Brinig and Allen 2000; Kalmijn and Poortman 2006). Because this decision often takes men by surprise (Thomas 1982), they might become more distressed when their marriage breaks down. Women who initiate divorce might already feel the relief of having terminated an unhappy relationship. These considerations suggest that men’s and women’s health and subjective well-being may adapt on different time scales: Women suffer from the impending end of a marriage already in predivorce years, whereas this process is delayed—and possibly more devastating—for men.

However, results regarding men’s greater vulnerability to the adverse effects of divorce on health and well-being outcomes are not consistent. Some studies have reported the opposite pattern (Aseltine and Kessler 1993; Simon and Marcussen 1999), and others have found no gender differences (Horwitz et al. 1996; Mastekaasa 1995; Strohschein et al. 2005). In view of this inconsistency, review articles have concluded that no compelling evidence exists to substantiate the claim that following a divorce, women are generally better off in terms of health and subjective well-being (Amato 2000; Amato and James 2010).

Another line of research on how divorce affects health and well-being has focused on mediating factors, such as changes in drinking, smoking, and body weight. Health behavior has been highlighted as a pivotal factor explaining why marriage benefits health and, conversely, why union dissolution harms health (Umberson et al. 2010). Married people drink and smoke less (Bachman et al. 2002; Chilcoat and Breslau 1996), but they also exercise less and weigh more (Grzywacz and Marks 1999; Jeffery and Rick 2002; The and Gordon-Larsen 2009). Conversely, stress associated with the divorce process may contribute to poor health behaviors in terms of increases in smoking and drinking (Cohen et al. 1991; Horwitz and White 1991), but it may also entail beneficial health effects in terms of weight loss. Regarding gender differences, extant research has shown that although men more often exhibit poor health behavior than women, changes across the divorce process do not differ in major ways (Umberson 1992).

### Social Outcomes of Divorce

Custodial arrangements represent the first and most intensely studied theme related to social outcomes of divorce. Noncustodial parents—usually fathers—face the challenge of maintaining contact with their children (Vogt Yuan 2014). Custodial parents—usually mothers—face the challenge of solo parenting and finding childcare (Goldberg et al. 1992). As a result, divorce is expected to have a negative effect on the quality of family life of both spouses (Umberson and Williams 1993). Research has suggested that fathers may suffer more than mothers in this domain (Leopold and Kalmijn 2016), particularly when they lose (or fear losing) contact with children (Bauserman 2012).

A second theme involves the chances of repartnering after divorce, commonly found to be higher among men. In the Netherlands, for example, 70 % of men and 50 % of women repartnered in the first 10 years after divorce (De Graaf and Kalmijn 2003). Men’s advantage in repartnering has also been found in other European countries (Ivanova et al. 2013) and in the United States (Wu and Schimmele 2005). Potential reasons for the gender difference in repartnering are threefold. First, people with resident children are less likely to repartner, and women more often get custody (Ivanova et al. 2013). Second, older people are less attractive on the remarriage market, and this age effect is stronger for women (Bennett 2017; Skopek et al. 2011). Third, people with fewer meeting opportunities are less likely to repartner, and women may be disadvantaged in terms of meeting opportunities in contexts such as the workplace (De Graaf and Kalmijn 2003).

A third theme comprises the consequences of divorce for social integration beyond the ties to partners and children. These consequences have been measured in terms of the number of friends; frequency of social participation; and frequency of contact with friends, relatives, and neighbors. According to the *liberation hypothesis* (Kalmijn and Broese van Groenou 2005), divorce promotes social integration in these areas because it terminates the dyadic withdrawal of couples. Moreover, divorce may increase the need for social contacts to compensate for the loss of a prime interaction partner and to get social support that helps in coping with the divorce process. According to the *isolation hypothesis* (Kalmijn and Broese van Groenou 2005), divorce entails not only the loss of a partner but also disruption of a shared social network and shared activities (Broese van Groenou 1991) as well as the loss of neighborhood ties in cases of residential moves. Moreover, these losses are not easily compensated for given that interaction partners as well as social settings allowing to form new ties are not readily available to many divorcees. These competing hypotheses are not explicitly gendered: their main arguments apply equally to men and women. An analysis of Dutch data supported the isolation hypothesis in most interaction domains, although contact with friends increased for women and particularly for men (Kalmijn and Broese van Groenou 2005). The study showed no major gender differences in the consequences of divorce, although effects on women appeared to be more strongly mediated by changes in resources. Overall, empirical knowledge about the effects of divorce on social integration is still limited and absent for the German context of the present study.

### Divorce in the West German Context

Because my analysis uses data from West Germany, it is important to understand specific historical, legal, and societal aspects of divorce. The sole ground for getting a divorce in Germany is disruption of a conjugal relationship beyond the point of restoration. When both spouses agree to a divorce, they can apply for a divorce after an obligatory year of separation. A divorcee can request spousal support, but maintenance claims are conditional on specific aspects of the preceding marriage, such as childcare, leave duration, and living standard. German maintenance law ensures a relatively high level of spousal support for economically dependent spouses and children, although more recent reforms have limited the period of entitlement to spousal maintenance (Bröckel and Andress 2015).

Germany has been described as a typical male breadwinner state (Lewis 1992), in which policy encourages men’s work in the market and women’s work in the home. This contrasts with the liberal tradition of U.S. policy that encourages women to invest in their human capital and to participate in the workforce. In Germany, taxation provides strong incentives to combine a breadwinner’s larger income with a homemaker’s smaller income, reinforcing a traditional division of labor during marriage (Cooke 2006). Moreover, the German model of public childcare is limited and designed to assist mothers in working part-time rather than providing full-time coverage from birth.

This context of a conservative male breadwinner model appears conducive to gender inequality in the effects of divorce, particularly regarding economic consequences for women. Reforms after the turn of the millennium have targeted some of these issues by implementing elements of the Nordic welfare model, including an expansion of public childcare, stronger economic incentives for mothers to return to the workforce, and other policies aimed at providing equal opportunities for men and women. The effects of these recent changes are still modest, although women’s labor force participation and use of childcare are on the rise (Bröckel and Andress 2015).

## Method

### Data and Sample

My analysis was based on data from 32 waves of the German SOEP (SOEP-long, version 32.1, release 2017; Wagner et al. 2007). For my purposes, these data yielded two main benefits. First, the SOEP includes multiple observations of respondents and short gaps between observations: data are available annually between 1984 and 2015. This large window of closely spaced observations allowed me to study gender differences across the divorce process. Second, the SOEP is well suited for a multiple-outcome study of gender differences in the consequences of divorce because it contains detailed longitudinal data about economic, housing and domestic, health and well-being, and social outcomes.

My aim was to offer a comprehensive view of gender differences in the consequences of divorce in terms of women’s and men’s year-to-year changes in multiple outcomes. Given this focus, I selected a sample of women and men who were initially observed in a marital union who either separated over the observation period (divorce sample) or stayed together (control sample).

I used the following restrictions to define the sample accordingly. First, I selected 36,631 individuals born in Germany and living in the Federal Republic of Germany before unification in 1989. This restriction to West German natives ensured that the sample was selected on comparable sociohistorical conditions as well as legal regulations surrounding divorce, eliminating heterogeneity in these contextual characteristics pertaining to the oversamples of East Germans and immigrants. Second, I constrained the sample to observations between ages 21 and 60 (*N* = 28,548 individuals). This restriction concentrated the analysis on the typical age range of divorce, and it reduced age heterogeneity in the life course profiles of the outcome measures. Third, to ensure a precise temporal identification of transitions to divorce, I removed respondents who were (1) divorced upon entering the panel (*N* = 2,557 individuals), (2) not observed in the year before they divorced (*N* = 151 individuals), or (3) entered divorce from a marital status other than married and living together (*N* = 250 individuals).

The remaining sample comprised two subsamples. The *divorce sample* included respondents (1) who were initially observed sharing a household in a marital union, (2) who divorced across the observation period, and (3) for whom the year of divorce could be determined by consecutive observations in the panel. The year of divorce was defined as the year of separation, although change of the legal status from married to divorced is often delayed by an obligatory year of separation before divorce. I removed observations outside an interval of 5 years before or after the year of divorce. This restriction ensured that I could draw on a sufficient number of observations across time points before and after divorce. After this exclusion, the divorce sample consisted of 1,222 individuals comprising 10,249 observations (person-years).

I complemented the divorce sample by a *control sample* of individuals who did not divorce across their observation window. I constrained the control sample to observations in which individuals were married and living together (*N* = 16,808 individuals comprising 127,003 observations). The benefits of keeping a control sample were twofold. First, observations from the control sample enabled me to better account for time-changing heterogeneity (e.g., age and period effects on the outcome measures) given that a much larger set of panel observations was available to estimate these effects. Second, a comparison between divorce sample and control sample provided information about compositional differences and selectivity, indicated by the extent to which the event sample differed from control sample in terms of the measures used in the analysis.

Table 1 presents descriptive information about the divorce sample and the control sample. Upon their first observation in the panel, respondents who went on to divorce were younger, less educated, more often living with children, more often unemployed, and in slightly worse health than the control sample of those who stayed married. Respondents from the divorce sample were also observed longer and less likely to drop out before the last interview in 2015. These differences were due to conditioning this sample on observing a divorce across the panel. Because a divorce often occurred several years after initial observation in a marital union, this condition implied that people who dropped out of the SOEP and people who entered the SOEP in more recent years were underrepresented in the divorce sample, relative to the control sample.

**Table 1:** Divorce sample and control sample

	Divorce Sample			Control Sample			
Measure	M	SD	*N*	M	SD	*N*	Description/Survey Question
Age							
Men	37.3	8.4	539	41.5	9.6	8,219	
Women	34.6	8.3	683	39.8	10.2	8,589	
Year of Observation							
Men	1997.5	8.9	539	2000.5	10.7	8,219	
Women	1998.2	9.0	683	2000.3	10.7	8,589	
Age at Divorce							
Men	41.0	8.5	539				
Women	38.3	8.5	683				
Year of Divorce							
Men	2001.2	8.7	539				
Women	2001.8	8.8	683				
Education							Level of education measured by the CASMIN^a^ classification
Not completed							In school (CASMIN = 0)
Men	0.0		535	0.0		8,118	
Women	0.0		673	0.0		8,485	
Lower							Intermediate general qualification or lower (CASMIN 1, 2, 4)
Men	0.11		535	0.07		8,118	
Women	0.20		673	0.16		8,485	
Intermediate							Basic/intermediate vocational qualification or general maturity certificate (CASMIN 3, 5, 6)
Men	0.64		535	0.60		8,118	
Women	0.59		673	0.58		8,485	
Higher							Lower or higher tertiary education (CASMIN 8, 7, 9)
Men	0.24		535	0.33		8,118	
Women	0.20		673	0.26		8,485	
Child in Household							At least one child younger than 15 living in the household: 0 = no, 1 = yes
Men	0.63		539	0.54		8,219	
Women	0.66		683	0.51		8,589	
Unemployed							Registered as unemployed: 0 = no, 1 = yes
Men	0.07		532	0.04		8,219	
Women	0.05		672	0.04		8,589	
Health Satisfaction							“How satisfied are you with your health?” 0 = completely dissatisfied, 10 = completely satisfied
Men	7.0	2.2	532	7.3	2.1	7,816	
Women	7.1	2.2	672	7.4	2.2	8,147	
Year of Panel Entry							First year in which a respondent was interviewed in the SOEP
Men	1993.0	9.6	539	1999.4	11.0	8,219	
Women	1994.0	10.0	683	1999.4	10.9	8,589	
Years Observed							Year of last interview minus year of first interview
Men	18.7	9.4	539	10.4	8.9	8,219	
Women	17.4	9.6	683	10.6	9.0	8,589	
Attrition							Respondent dropped out of the panel before the most recent wave of 2015
Men	0.48		539	0.62		8,219	
Women	0.49		683	0.60		8,589	

*Note:* All statistics for time-changing variables are calculated for respondents’ first observation in the panel.

*Source:* SOEP, v32.1, release 2017.

^a^CASMIN = Comparative Analysis of Social Mobility in Industrial Nations.

### Outcome Measures

In Tables 2 and 3, I present descriptive statistics and detailed information about the measurement of all outcomes. I consider a total of 20 outcomes: (1) four economic outcomes covering objective and subjective aspects of economic status; (2) four housing and domestic outcomes covering residential moves, homeownership, and subjective and objective aspects of domestic work; (3) six health and well-being outcomes covering measures of mental health, physical health, general well-being, and health behaviors; and (4) six social outcomes covering objective aspects (union status, parenting status, and the frequency of visits to friends and relatives) and subjective aspects (satisfaction with family life and feelings of loneliness).

**Table 2:** Economic outcomes, health and well-being outcomes

	Divorce Sample			Control Sample			
Measure	*M*	SD	*N*	*M*	SD	*N*	Description/Survey Question
Economic Outcomes							
Satisfaction with standard of living							Measured 1991–1993, 1995–2006, and 2013.
Men	7.1	1.8	278	7.6	1.6	2,886	“How satisfied are you with your overall standard of living?”
Women	6.9	2.0	343	7.8	1.7	2,968	0 = completely dissatisfied, 10 = completely satisfied.
Satisfaction with income							Measured annually 1984–2015.
Men	6.1	2.4	531	6.7	2.2	7,784	“How satisfied are you with your household income?”
Women	6.2	2.4	671	7.0	2.2	8,112	0 = completely dissatisfied, 10 = completely satisfied.
Annual household income							Measured annually 1984–2015. Annual postgovernment household income calculated by the SOEP as the sum of total family income from labor earnings, asset flows, retirement income, private transfers, public transfers, and social security pensions minus family taxes. Private transfers include alimony and child support payments. Public transfers include housing allowances, child benefits, subsistence assistance, and maternity benefits (Grabka 2013). Adjusted for inflation (reference year 2011) and equivalized by square root scale.
Men	22,519	11,318	538	24,923	14,114	8,213	
Women	22,410	12,061	683	24,843	14,090	8,583	
Poverty							Measured annually 1984–2015. Indicator for whether annual postgovernment household income (see above) was < 60 % of the median value calculated for the respective survey year on the basis of the full SOEP sample of West Germans (European Commission definition of poverty).
Men	.12		538	.08		8,213	
Women	.14		683	.08		8,583	
Health and Well-being Outcomes							
Satisfaction with life							Measured annually 1984–2015. “How satisfied are you with your life, all things considered?” 0 = completely dissatisfied, 10 = completely satisfied.
Men	7.0	1.9	532	7.7	1.6	7,851	
Women	7.1	1.8	671	7.8	1.6	8,170	
Mental health (MCS)							Measured biannually 2002–2014. Based on a multi-item scale of the SF-12v2 questionnaire (Fleishman et al. 2010) that evaluates eight domains of functioning and well-being. Mental Component Scale (MCS) gives weight to mental health, social functioning, and emotional problems. Score calculated by the SOEP group for the reference year 2004 with *M* = 50 and SD = 10.
Men	50.3	7.4	80	52.0	8.7	933	
Women	50.2	9.0	100	50.9	9.3	981	
Physical health (PCS)							Measured biannually 2002–2014. Based on a multi-item scale of the SF-12v2 questionnaire (Fleishman et al. 2010) that evaluates eight domains of functioning and well-being. Physical Component Scale (PCS) gives weight to physical functioning, role limitations due to physical problems, and bodily pain. Score calculated by the SOEP group for the reference year 2004 with *M* = 50 and SD = 10.
Men	53.7	8.5	80	53.6	8.4	933	
Women	51.8	10.7	100	52.3	8.7	981	
BMI							Measured biannually 2002–2014. Weight (in kilos) divided by squared height (in meters).
Men	26.3	4.3	81	26.6	4.1	941	
Women	24.6	5.1	103	24.4	4.5	973	
Smoking							Measured biannually 2002–2014. Indicator for whether respondent currently smoked.
Men	.56		73	.34		899	
Women	.47		91	.25		943	
Drinking							Measured 2006, 2008, and 2010. Indicator for whether respondent regularly consumed beer and/or wine/champagne and/or spirits. Answer categories: regularly, once in a while, rarely, never.
Men	.28		43	.26		450	
Women	.12		50	.08		490	

*Note:* All statistics for time-changing variables are calculated for respondents’ first observation in the panel.

*Source:* SOEP, v32.1, release 2017.

**Table 3:** Housing and domestic outcomes, social outcomes

	DivorceSample			Control Sample			
Measure	*M*	SD	*N*	*M*	SD	*N*	Description/Survey Question
Housing and Domestic Outcomes							
Residential moves							Measured annually 1984–2015. Indicator for whether a respondent moved into the current housing unit in the current or previous calendar year.
Men	.21		539	.15		8,219	
Women	.18		683	.14		8,589	
Homeownership							Measured annually 1984–2015. Indicator for whether a respondent was the owner of the currently occupied housing unit.
Men	.40		539	.55		8,218	
Women	.38		683	.56		8,588	
Satisfaction with housework							Measured annually 1984–1990 and 1993–2015. “How satisfied are you with your work in the home?” 0 = completely dissatisfied, 10 = completely satisfied.
Men	6.5	2.2	301	7.1	2.1	4,195	
Women	6.4	2.2	601	7.0	2.0	7,491	
Hours of housework							Measured annually 1991–2012. “What does a typical weekday look like for you? How many hours do you spend on the following activities: Washing, cooking, cleaning?” Top-coded to 10 hours.
Men	0.8	0.8	371	0.7	0.8	5,696	
Women	3.0	1.8	504	2.9	1.7	6,098	
Social Outcomes							
Partner							Measured annually 1984–2015. Indicator for whether a respondent lived with a partner in the household.
Men	1		593	1		8,216	
Women	1		683	1		8,586	
Single parenting							Measured annually 1984–2015. Indicator for whether a respondent lived in a single-parent household.
Men	0		593	0		8,218	
Women	0		683	0		8,588	
Satisfaction with family life							Measured annually 2006–2015. “How satisfied are you with your family life?” 0 = completely dissatisfied, 10 = completely satisfied.
Men	7.6	2.0	114	8.6	1.5	3,130	
Women	7.6	2.1	169	8.6	1.4	3,243	
Loneliness							Measured in 1992, 1993, 1995, 1996, 1997, 2008, and 2013. “To what extent do you agree with the following statement? I often feel lonely.” Answers “completely agree” and “agree” coded 1, “disagree” and “completely disagree” coded 0.
Men	.11		84	.04		394	
Women	.16		103	.11		378	
Visiting relatives							Measured in 1990, 1995, 1998, 2003, 2008, and 2013. At least weekly visits coded 1, less than weekly visits coded 0.
Men	.56		70	.54		577	
Women	.45		91	.59		591	
Visiting friends							Measured in 1990, 1995, 1998, 2003, 2008, and 2013. At least weekly visits coded 1, less than weekly visits coded 0.
Men	.49		70	.51		578	
Women	.57		90	.58		593	

*Note:* All statistics for time-changing variables are calculated for respondents’ first observation in the panel.

*Source:* SOEP, v32.1, release 2017.

The data shown in Tables 2 and 3 pertain to every respondent’s first observation in the panel. For the divorce sample, this observation predates the separation of a union by at least 1 year (see the aforementioned sample selection criteria). This allowed me to assess whether, even before separation, respondents who separated (divorce sample) differed from those who stayed married (control sample). However, predivorce differences between the divorce sample and the control sample may reflect selection into divorce (e.g., unhappier, poorer, and unhealthier individuals being more likely to separate) as well as the influence of impending divorce given that divorce is most commonly experienced as a process rather than as a sudden event.

The descriptive statistics on the first panel observation presented in Tables 2 and 3 indicate that compared with women and men who stayed married, those who went on to divorce were less satisfied with life, family life, income, housework, and their standard of living. Respondents from the divorce sample also earned approximately 10 % less, were more likely to live below the poverty line, and were less likely to own their home. Furthermore, future divorcees showed lower mental health and similar physical health and body mass index (BMI) compared with those who would stay married. Large differences of more than 20 percentage points were found for smoking behavior, with respondents from the divorce sample smoking more often than their counterparts who stayed married. Differences in drinking were much smaller. Finally, social integration with friends and relatives was similar for the control sample and the divorce sample, but respondents from the latter group were more likely to experience feelings of loneliness.

### Measures of the Divorce Process

To assess changes across the divorce process, I modeled all outcomes as linear functions of time before and after divorce. I allowed for variation in the effects of time, captured by a set of dummy variables designating five periods: (1) 5 to 3 years before divorce (reference period), (2) 2 to 1 years before divorce, (3) year of divorce, (4) 1 to 2 years after divorce, and (5) 3 to 5 years after divorce. These measures jointly represented the effect of time on the outcome measures, allowing me to study changes before and after divorce. I assessed divorce effects relative to all observations in a marital union (i.e., the divorce sample’s observations in the reference period and the control sample’s continued observations in a marital union). Respondents from the control sample did not enter into the estimation of divorce effects, but they contributed to identifying the effects of the control variables.

### Controls

Given the time dependency of divorce effects, I controlled for life course profiles (changes with age) and the periodic profiles (changes across calendar years) of the outcomes. Age effects and period effects might introduce bias in the estimation of temporal profiles of change in the outcomes across predivorce and postdivorce stages. For example, if the age effect on subjective well-being is negative, an uncontrolled model could overestimate initial drops and underestimate subsequent adaptation. To break collinearity between the divorce indicators and the controls, I included age and period in categorical form, each capturing change across 4-year intervals. Additional analyses (not shown) showed that the results were robust to changes in the span of these categories (e.g., using categorical variables for 3-year intervals) and in the parametrization of the age effects (e.g., replacing age categories by linear, squared, cubic, and quartic age terms).

Adverse events that can trigger the divorce process and influence the outcomes of interest represented another potential source of bias. Two such factors that have been examined in the literature are job loss (Dorion and Mendolia 2012) and poor health (Blekesaune and Barrett 2005). Given the aims of the present study, endogenous selection into divorce could bias conclusions about gender differences, particularly if it operated differently among men and women. Research has shown that these concerns may be warranted given that the effects of job loss on well-being were found to be stronger for men than for women (Leopold et al. 2017).

To address this source of bias, I added two time-varying controls to my models. First, I included an indicator variable for whether a respondent was registered as unemployed. Second, I controlled for a respondent’s satisfaction with health. Health satisfaction, measured on an 11-point Likert scale at every panel wave, is a valid and reliable health measure that is highly correlated with other measures of self-rated health and predictive of objective outcomes such as mortality (Idler and Benyamini 1997).

Although it was important to control for these experiences *before* divorce, canceling out their effects *after* divorce would be undesirable because both factors could mediate divorce effects on several of the outcomes under consideration (see Amato 2000:1272). For example, the effect of divorce on subjective well-being might partly run through declines in health satisfaction. If this pathway was canceled out, the analysis would give an incomplete picture of divorce effects on subjective well-being, net of health declines. Similarly, the effect of divorce on the risk of poverty might partly run through job loss related to the divorce crisis and associated residential moves. If this pathway was canceled out, the analysis would give an incomplete picture of divorce effects on the risk of poverty, net of the risk of job loss as a potential mediator of such effects. To avoid overcontrolling in postdivorce periods, I specified the controls for unemployment and health satisfaction as to account for endogenous selection into divorce but not for postdivorce changes in the outcomes. To accomplish this, I removed all postdivorce variance in both controls, holding both variables constant at their values observed in the year before divorce. In the control sample, no adjustment was made.

### Model

To estimate change in the outcome measures, I used fixed-effects linear regression models. Changes in binary outcome measures were estimated by fixed-effects linear probability models. Fixed-effects models focus only on changes within individuals over time, relating temporal variation in the outcome measures only to temporal variation in the independent variables. Because only characteristics that vary over time can enter the fixed-effects model, all time-constant variables drop out of the equation. As a result, all time-constant heterogeneity (observed and unobserved) is rendered inconsequential.

**Fig. 1:**
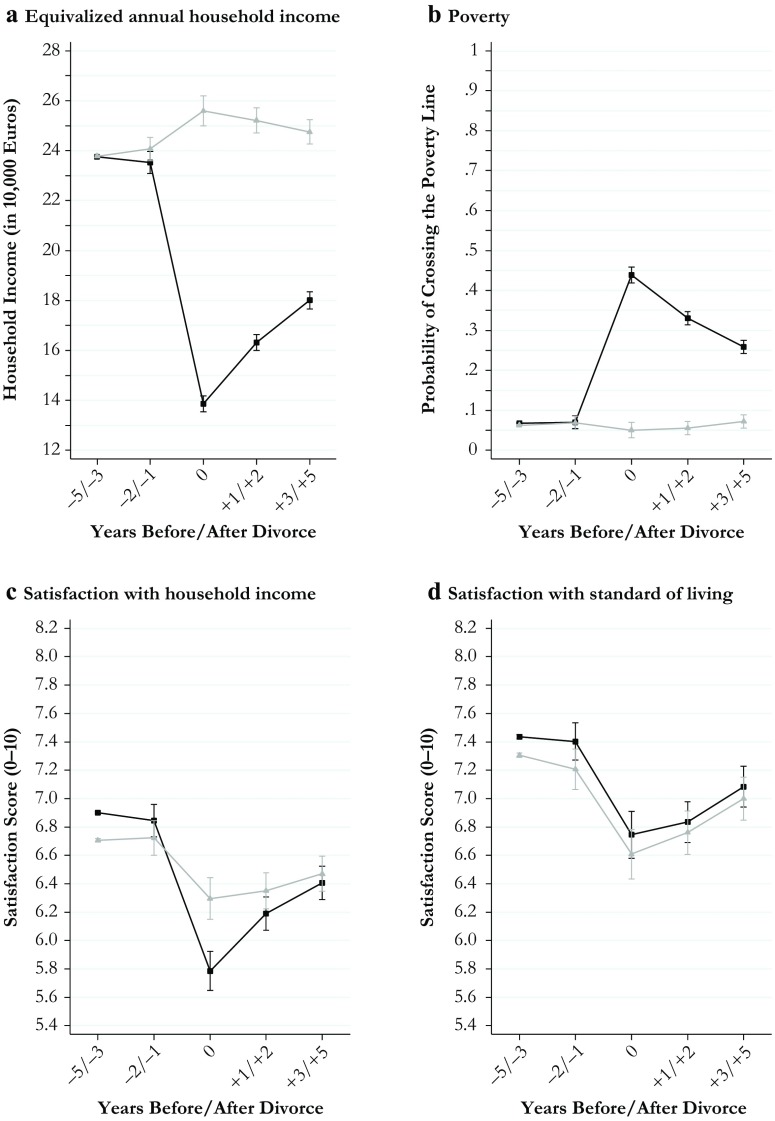
Economic outcomes of divorce: Women (black curves) and men (gray curves)

**Fig. 2:**
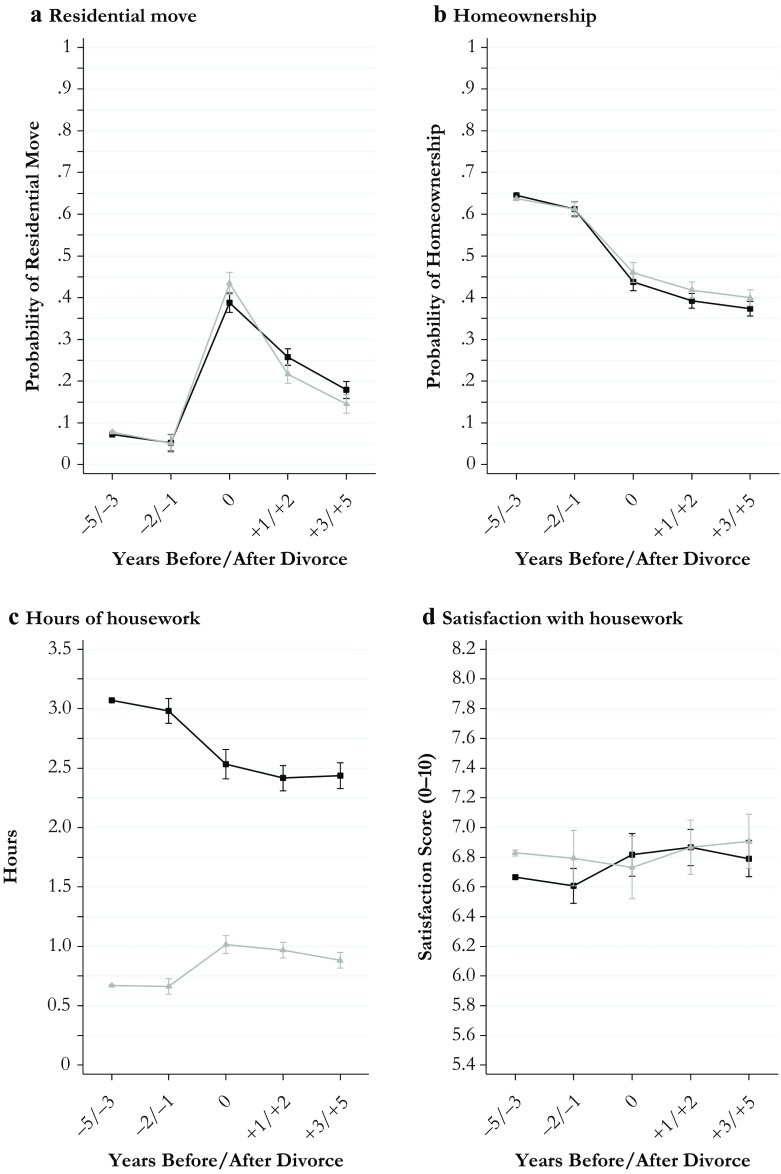
Housing and domestic outcomes of divorce: Women (black curves) and men (gray curves)

**Fig. 3:**
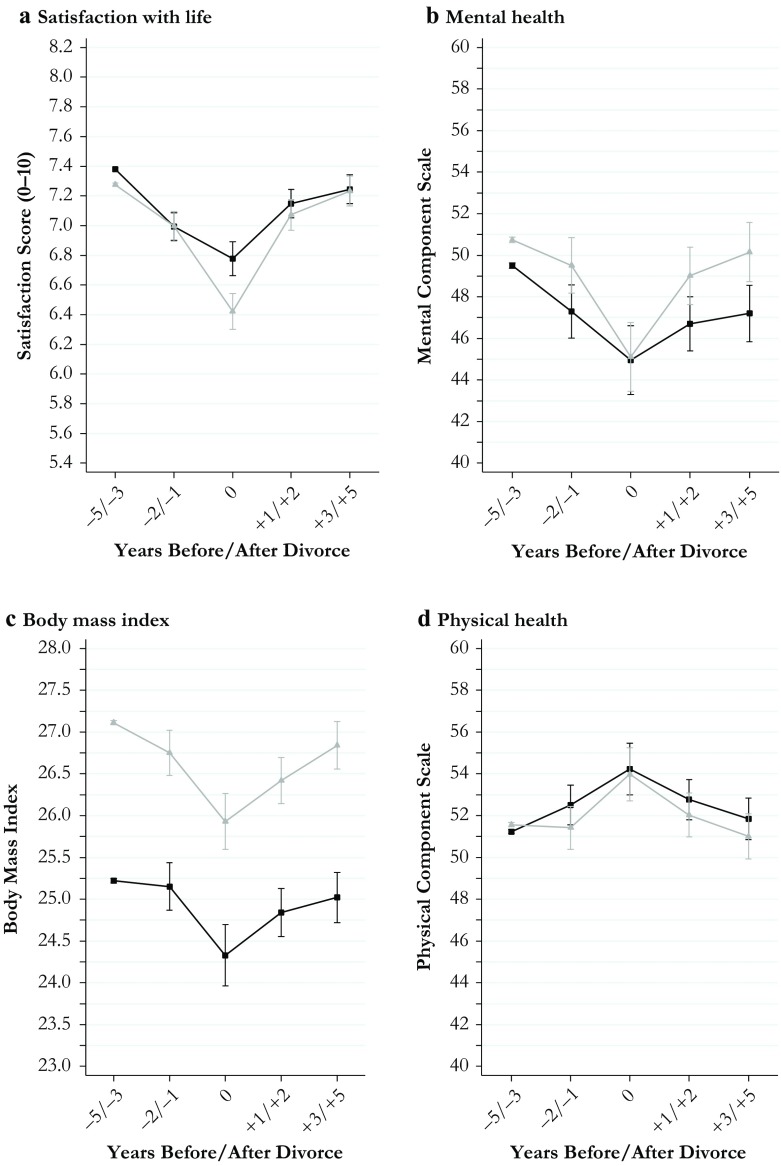

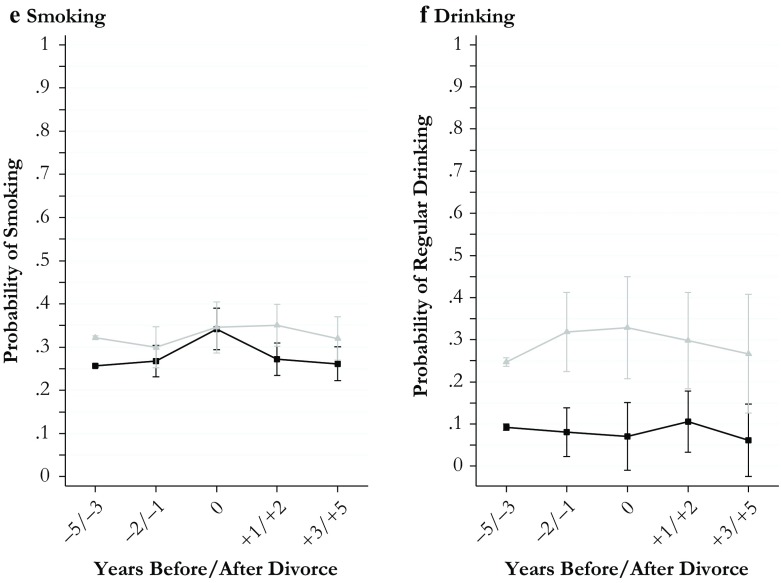
Health and well-being outcomes of divorce: Women (black curves) and men (gray curves)

**Fig. 4:**
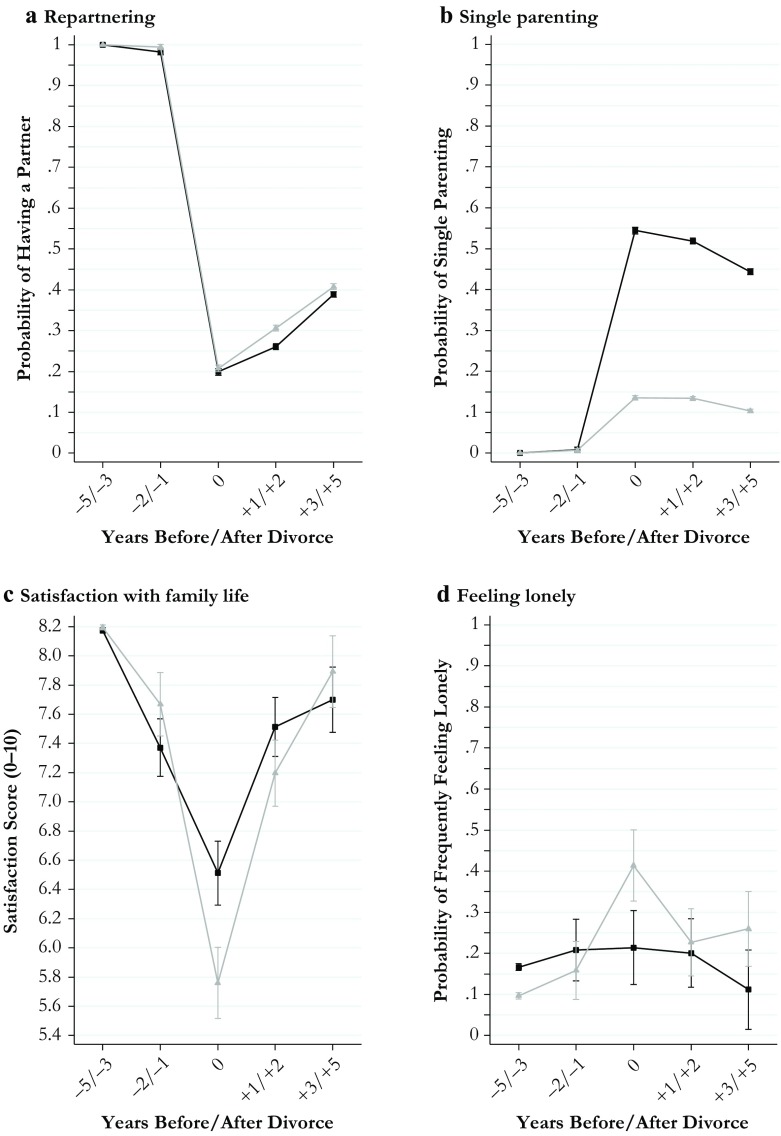

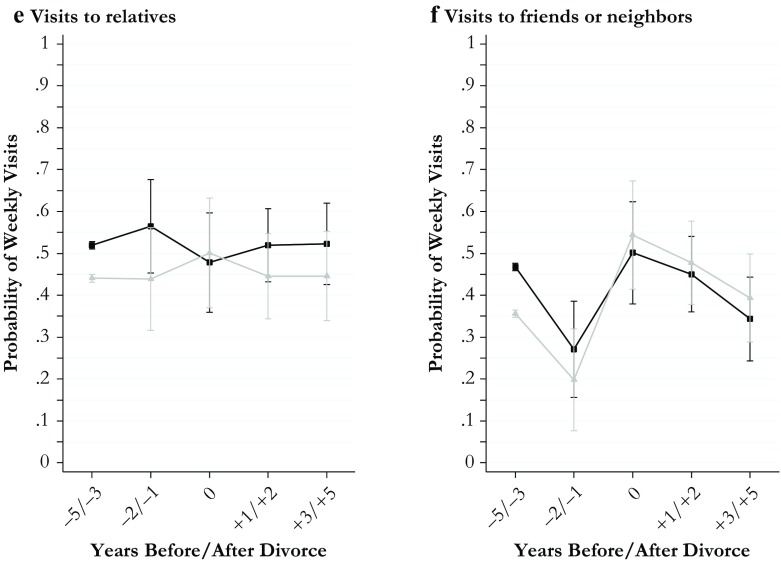
Social outcomes of divorce: Women (black curves) and men (gray curves)

I estimated all models separately for men and women to keep the model parsimonious and to retain information about gender differences in the level of the outcomes estimated for the reference period. All estimates for divorce effects obtained from these models along with their 95 % confidence intervals are shown in Figs. 1–4. The models behind the plots are detailed in Tables S1–S4 in Online Resource 1. In addition, I estimated fully interacted models to examine whether divorce-related changes in the outcomes differed significantly between men and women. The interactions between the divorce indicators and gender estimated from fully interacted models are shown in Tables S5 and S6, Online Resource 1. Because of the large number of statistical tests performed in my models, I used strict criteria (*p* < .01 and *p* < .001) to evaluate statistical significance.

Finally, I examined whether inclusion of the interactions between the divorce indicators and gender improved model fit in the fully interacted models. Because my interest was in changes in explained variance *within* individuals over time (“within-*R*^2^”), I compared the fit of these nested models specified as ordinary least squares (OLS) linear regression models including dummy variables for each individual, a method that yields estimates identical to within-transformed fixed-effects estimates. The results on changes in model fit for each outcome are summarized in Table S7, Online Resource 1.

## Results

The plots presented in Fig. 1 (economic outcomes), Fig. 2 (housing and domestic outcomes), Fig. 3 (health and well-being outcomes), and Fig. 4 (social outcomes) illustrate change in all 20 outcome measures across the divorce process separately for women (black curves) and men (gray curves). Average marginal effects are shown for fixed values of the divorce indicators.

### Results for Economic Outcomes

Figure 1 shows gender differences in the consequences of divorce for four economic outcomes. Panel a illustrates the scope of postdivorce gender inequality in equivalized household income. In the year of divorce, women lost approximately 40 % of their predivorce incomes, whereas their former husbands experienced moderate gains of approximately 5 %. In subsequent years, women’s incomes recovered to reduce the resulting average gender gap from more than 11,000 Euros to approximately 6,500 Euros of equivalized annual household income (all income listed in 2011 values). These gendered shifts in economic status were also reflected in the second outcome measure indicating the probability of crossing the poverty line (i.e., having less than 60 % of year-specific median household income; panel b of Fig. 1). Women’s poverty risk surged upward in the year of divorce. For this year, linear probability models estimated a sixfold increase from a predivorce risk of approximately 7 % to almost 45 %. Although women recovered in subsequent years, their poverty risk remained above 25 % even several years after divorce. Men’s poverty risk remained unchanged across the divorce process.

How did women and men experience these changes subjectively? For the measure of satisfaction with household income, I found that women experienced larger declines than men in all postdivorce years compared with the predivorce reference period (*p* < .001; Table S5). Panel c of Fig. 1 shows, however, that despite these disproportionate losses, women’s average satisfaction with their household income dropped below men’s averages only in the year of divorce. Three to 5 years after divorce, average gender differences in satisfaction with household income were reduced almost to 0. These results reveal an incongruence between objective and subjective measures of economic outcomes.

Looking at a broader measure of satisfaction with the overall standard of living, I found no significant gender differences in the magnitude of declines across the divorce process (Table S5). Women’s levels remained above men’s levels across the entire divorce process (Fig. 1, panel d). In contrast to the measures for income and poverty, these results on the subjective measures of economic well-being indicated smaller and transient gender differences.

### Results for Housing and Domestic Outcomes

Figure 2 illustrates the consequences of divorce for housing and domestic outcomes. Panel a shows that men were slightly more likely to move in the year of divorce and that women were more likely to move in the following years. However, gender differences in the effects of divorce on the probability of residential moves were relatively small and were significant only for women’s higher probability of moving 1 to 2 years after divorce (Table S5). A similar result of small gender differences emerged for divorce-related declines in homeownership (panel b, Fig. 2). These medium-term drops amounted to more than 20 percentage points among both women and men; gender differences were insignificant, although declines were slightly steeper for women (Table S5).

I found a contrasting pattern of large and highly significant gender differences for changes in hours of routine housework (panel c, Fig. 2). Women performed approximately one-half hour less following divorce, whereas men’s daily housework time increased by approximately 20 minutes. The resulting gender convergence in housework time was permanent, although a large gap remained in postdivorce years. In subjective terms, women’s and men’s satisfaction with housework did not change in meaningful ways across the divorce process, although an indication for a slight relief effect was found among women in postdivorce years (panel d).

### Results for Health and Well-being Outcomes

Figure 3 illustrates the consequences of divorce for six measures of health, health behaviors, and well-being. The overall pattern of results is notable for the absence of major gender differences (Table S5). The only larger and statistically significant difference was that men’s initial declines in life satisfaction exceeded those of women (panel a, Fig. 3). Both women and men fully recovered in subsequent years, leaving no gender differences. Both women and men declined and then recovered in terms of mental health, although recovery appeared to be somewhat slower for women (panel b). Both women and men lost and then regained weight (panel c), and both improved slightly in terms of physical health and then declined toward predivorce levels (panel d). Finally, both women and men changed little in their smoking and drinking habits (panels e and f). None of the gender gaps in terms of health and well-being outcomes changed in meaningful ways when predivorce and postdivorce periods are compared.

### Results for Social Outcomes

Figure 4 relates to my last set of outcomes, pertaining to the social consequences of divorce. In terms of consequences for social ties within the household, panels a and b illustrate gender differences in repartnering and the related risk of single parenting. Regarding repartnering, my findings were consistent with earlier research showing higher chances of repartnering among men. Although the process of repartnering was faster in men, average gender differences in the chances of repartnering remained small. In the final period studied (3 to 5 years after divorce), approximately 40 % of men and slightly less than 40 % of women were living with a partner. In terms of the related risk of single parenting, a large gender gap of approximately 40 percentage points (55 % of women vs. 14 % of men) opened up in the year of divorce and did not change much in subsequent years.

Looking at the consequences of divorce for social ties outside the household, I found few gender differences. Women’s and men’s frequency of visits to relatives remained constant throughout the study period (panel e, Fig. 4), whereas the frequency of visits to friends and neighbors was more responsive to the divorce process (panel f). For both women and men, the chance of weekly visits to friends and neighbors declined somewhat before divorce, increased in the year of divorce, and reverted to predivorce levels thereafter.

Finally, I assessed how these changes were experienced subjectively, measured by indicators for satisfaction with family life and feelings of loneliness. Satisfaction with family life showed the strongest reactions to the divorce process among all satisfaction measures examined in this study (panel c, Fig. 4). This applied particularly to men who experienced average drops of 2.5 scale points between the reference period and the year of divorce. The magnitude of this effect amounted to 2 standard deviations of within-person variation in satisfaction with family life measured in the full sample of the SOEP. I found sizable but significantly smaller drops for women. The resulting gender gap in satisfaction with family life peaked in the year after divorce: women were favored by almost 1 scale point. In subsequent years, the gap narrowed and vanished in the period of 3 to 5 years after divorce. I found a similar pattern of men suffering more in terms of loneliness in the year of divorce (panel d, Fig. 4). More than 40 % of men reported frequent or very frequent feelings of loneliness in this year, approximately double the share of women who felt lonely. The gender gap in loneliness narrowed over the next years, although increases in men’s levels remained significantly larger than changes in women’s levels in the medium term.

## Discussion

Divorce affects various aspects of health and psychological well-being as well as economic, social, and domestic life. Research on gender differences in the consequences of divorce has typically focused on only one of these domains. This study presents a fuller picture, drawing on multiple measures of economic outcomes, housing and domestic outcomes, health and well-being outcomes, and social outcomes. To examine gender differences in the consequences of divorce in the short term and in the medium term, I examined changes in these measures over a period of up to 5 years before and after divorce.

Three main findings emerged from the analysis. First, a medium-term view on multiple outcomes yielded an overall picture of similarity, rather than differences, between women and men. Women and men did not differ much in terms of the consequences of divorce for (1) subjective economic well-being; (2) residential moves, homeownership, and satisfaction with housework; (3) mental health, physical health, and psychological well-being; and (4) chances of repartnering and social integration with friends and relatives. These findings on the absence of clear-cut gender differences are consistent with previous research on similar measures, including studies on subjective economic well-being (Andress and Bröckel 2007), health and psychological well-being (Strohschein et al. 2005), residential moves (Feijten and Mulder 2010; Mulder and Malmberg 2011) and homeownership (Dewilde and Stier 2014), and social integration (Kalmijn and Broese van Groenou 2005; Kalmijn and Uunk 2006).

Second, where gender differences emerged, they were mostly short-lived. Men experienced larger drops in satisfaction with life and particularly in satisfaction with family life observed in the year of divorce, but over the next years, the gender gap in these outcomes vanished. The same pattern was observed for women’s larger declines in satisfaction with household income, suggesting that gender differences in the consequences of divorce are generally larger in the short term than in the medium term.

Taken together, these findings on the absence of gender differences seem to contradict theoretical considerations about several outcome measures under consideration in the present study. One potential reason for this is that many of these considerations allude to countervailing mechanisms that may offset each other when measuring average changes across the divorce process in a larger population male and female divorcees. In the case of residential moves, for example, women may more often leave the shared household for economic reasons, whereas men may more leave the shared household for family reasons related to child custody. Similarly, if women’s coping is more internalized and men’s coping more externalized, the negative effects of both stress responses on general health measures may not differ much, on average. A further potential reason for the absence of medium-term gender differences in many outcomes is adaptation. This tendency of returning to predivorce levels after some years, alleviating gender differences in the process, is considered to be a universal force that does not differ by gender except for specific circumstances, such as unemployment (Clark et al. 2008).

Third, I found large gender differences for a few of the 20 outcome measures. Most notably, women were strongly disadvantaged in terms of losses in household income and associated increases in the risk of poverty. Moreover, women’s disproportionate losses in these objective measures of economic status were permanent. Although the gender gaps in household income and risk of poverty narrowed somewhat over time, differences between women and men remained substantial. The same applied to single parenthood.

Looking at the big picture of knowledge about gender differences in the effects of divorce, these conclusions demonstrate the benefit of considering multiple outcomes in the analysis. This applies not only to the coverage of different domains in which divorce effects unfold but also to the inclusion of objective and subjective measures. For example, gender gaps looked different depending on whether objective economic status or subjective economic well-being was examined. This distinction is important for theories of the divorce process, given that the crisis model and the chronic strain model highlight subjective factors, such as the actual distress that individuals experience.

Confidence in the results for subjective measures of satisfaction in different domains of life is strengthened by research showing that the single-item measures used in this study are sensitive, valid, and reliable (Diener et al. 2013; Hazelrigg and Hardy 1999; Schwarze et al. 2000; Veenhoven 1996). The incongruence found between gender differences in objective economic status and subjective measures of economic well-being speaks to a long-standing tradition of research on the quality of life (Campbell et al. 1976), emphasizing that objectively good or bad conditions are not necessarily experienced as such. Women, for example, may initially feel deprived when comparing their predivorce and postdivorce incomes but then adjust their frame of reference over time. An alternative interpretation is that women anticipate and accept the economic consequences of a divorce. This would also explain why women more often initiate a divorce despite the expectation of disproportionate economic losses (Andress and Bröckel 2007:501).

My results support a number of specific ideas that have been advanced in previous research about gender differences in the consequences of divorce. The measure of life satisfaction, for example, indicated that women’s and men’s subjective well-being adapted on different time scales. The temporal pattern found is consistent with the idea that separation brings relief to women whereas it exacerbates distress among men (Andress and Bröckel 2007; Thomas 1982). It also mirrors the finding that women are more likely to initiate divorce than men (Kalmijn and Poortman 2006).

In the domestic sphere, the measure of satisfaction with family life was consistent with the finding that the noncustodial parent suffers more than the custodial parent after a divorce (Bauserman 2012). Finally, the findings on changes in housework were in line with the idea that the division of labor becomes less gendered after marital dissolution (Gupta 1999; Hewitt et al. 2013). The finding of a converging gender gap is in line with other studies showing that although the division of labor is mostly stable across the life course, key transitions such as parenthood, divorce, and retirement lead to substantial and permanent changes (Gupta 1999; Kühhirt 2012; Leopold and Skopek 2015).

Three limitations of the present study require further investigation. First, the data did not include sufficient longitudinal information to assess gender gaps in more objective measures of health, such as cortisol levels and other biomarkers. My measures of health behavior were also limited—particularly data about drinking behavior that were available for only a few panel waves and did not directly measure alcohol abuse. The latter omission is important given that research has indicated that men are more likely to exhibit externalizing behavior in reaction to stress (Horwitz and Davies 1994).

Second, the results found for medium-term adaptation eliminating initial gender differences might at least partly reflect selective attrition. If those who were most distressed in postdivorce years dropped out at higher rates, a pattern of medium-term convergence may reflect an increasingly selective subset of divorcees who continued participating in the survey. In my analytic sample, attrition rates were lower among divorcees than among those who stayed married. These differences suggest that those who participate long enough to observe a divorce (i.e., at least once after separation) are more reliable respondents with lower baseline probabilities of exiting the panel. With regard to gender differences, rates of attrition were nearly identical for men and women. Although these results alleviate some of the concerns associated with selective attrition, respondents who dropped out shortly before or after a divorce are underrepresented in my sample. If these divorces are especially painful and their consequences are more strongly gendered, my findings might still be tilted toward more peaceful instances of “clean breakups.”

Third, my conclusions are limited to the West German context from which the data were drawn. As noted, Germany is an interesting setting to examine gender differences in the consequences of divorce because it has long represented an ideal type of a male breadwinner state. This model is conducive to gender inequality in the economic impact of marital disruption, and the economic domain was the key area in which large and persistent gender gaps emerged. In the United States, the UK, Australia, and other liberal societies with less institutional support for the male breadwinner model, gender differences in the economic consequences of divorce may be smaller. A recent study showed that compared with Germany, women’s short-term and medium-term losses in household income were indeed smaller in the UK and in Australia but not in the United States (de Vaus et al. 2015). Given the lack of comparative studies on larger sets of outcomes, broader conclusions about cross-national variation in the gendered consequences of divorce require further multiple-outcome studies using data from other national contexts.

Returning to the opening question of this article, my findings suggest that the prevailing view of women bearing a higher burden of divorce is supported when looking at medium-term consequences for a large set of outcome measures, including those on which men were previously found to be disadvantaged. Taking economic, housing and domestic, health and well-being, and social outcomes into account, men were more vulnerable to short-term effects on subjective measures of well-being, but women experienced medium-term disadvantages in objective economic status. In other words, men’s disproportionate psychological strain was transient, whereas women’s disproportionate economic strain was chronic.

## Electronic supplementary material

ESM 1 (PDF 436 kb)
