# Supplementary material for: Gender Differences in the Consequences of Divorce: A Study of Multiple Outcomes
Source: Demography. 2018 Apr 13;55(3):769–97. doi: 10.1007/s13524-018-0667-6 (PMC5992251; doi:10.1007/s13524-018-0667-6)
Supplement: Supplementary file 1 — (PDF 436 kb) [file 13524_2018_667_MOESM1_ESM.pdf]

Online Resource 1

Gender Differences in the Consequences of Divorce: A Study of Multiple Outcomes

Thomas Leopold

Table S1. *Fixed-Effects Models for Economic Outcomes*

	Annual Household Income (log)		Risk of Poverty		Satisfaction with Income		Satisfaction with Standard of Living	
	Women	Men	Women	Men	Women	Men	Women	Men
Years before/after divorce (ref.: -3/-5)								
-2/-1	-0.01	0.01	0.00	0.01	-0.06	0.02	-0.03	-0.10
	(0.01)	(0.01)	(0.01)	(0.01)	(0.06)	(0.06)	(0.07)	(0.07)
0	-0.54***	0.07***	0.37***	-0.01	-1.12***	-0.41***	-0.69***	-0.70***
	(0.01)	(0.01)	(0.01)	(0.01)	(0.07)	(0.08)	(0.09)	(0.09)
1/2	-0.38***	0.06***	0.26***	-0.01	-0.71***	-0.36***	-0.60***	-0.54***
	(0.01)	(0.01)	(0.01)	(0.01)	(0.06)	(0.07)	(0.08)	(0.08)
3/5	-0.28***	0.04***	0.19***	0.01	-0.50***	-0.24***	-0.35***	-0.31***
	(0.01)	(0.01)	(0.01)	(0.01)	(0.06)	(0.07)	(0.08)	(0.08)
Age (ref.: 21-24)								
25-28	0.02	0.02	-0.02**	-0.02	0.14**	0.20	-0.19**	-0.14
	(0.01)	(0.01)	(0.01)	(0.01)	(0.06)	(0.09)	(0.07)	(0.11)
29-32	0.02	0.01	-0.03***	-0.03**	0.23***	0.40***	-0.24**	-0.13
	(0.01)	(0.01)	(0.01)	(0.01)	(0.06)	(0.09)	(0.08)	(0.12)
33-36	0.03**	0.01	-0.05***	-0.04**	0.34***	0.47***	-0.29**	-0.20
	(0.01)	(0.02)	(0.01)	(0.01)	(0.07)	(0.10)	(0.09)	(0.12)
37-40	0.09***	0.04	-0.07***	-0.05***	0.43***	0.60***	-0.29**	-0.19
	(0.01)	(0.02)	(0.01)	(0.01)	(0.08)	(0.10)	(0.10)	(0.13)
41-44	0.16***	0.09***	-0.08***	-0.06***	0.44***	0.65***	-0.35**	-0.26
	(0.01)	(0.02)	(0.01)	(0.02)	(0.09)	(0.11)	(0.11)	(0.14)
45-48	0.25***	0.16***	-0.10***	-0.06***	0.46***	0.65***	-0.40**	-0.37*
	(0.02)	(0.02)	(0.01)	(0.02)	(0.10)	(0.12)	(0.13)	(0.16)
49-52	0.31***	0.23***	-0.10***	-0.07***	0.47***	0.66***	-0.48***	-0.51**
	(0.02)	(0.02)	(0.02)	(0.02)	(0.11)	(0.13)	(0.14)	(0.17)
53-56	0.30***	0.27***	-0.08***	-0.06**	0.50***	0.72***	-0.55***	-0.61***
	(0.02)	(0.02)	(0.02)	(0.02)	(0.12)	(0.14)	(0.16)	(0.18)
57-60	0.24***	0.25***	-0.05	-0.04	0.53***	0.77***	-0.64***	-0.64**
	(0.02)	(0.02)	(0.02)	(0.02)	(0.14)	(0.15)	(0.17)	(0.19)
Period (ref.: 1984-1987)								
1988-1991	0.11***	0.12***	-0.01	-0.01	0.17***	0.25***		
	(0.00)	(0.00)	(0.00)	(0.00)	(0.03)	(0.03)		
1992-1995	0.17***	0.19***	-0.00	-0.01	0.03	0.21***	-0.10**	-0.06
	(0.01)	(0.01)	(0.01)	(0.01)	(0.04)	(0.04)	(0.04)	(0.04)
1996-1999	0.12***	0.15***	-0.01	-0.03***	-0.08	0.04	-0.12**	-0.06
	(0.01)	(0.01)	(0.01)	(0.01)	(0.05)	(0.05)	(0.05)	(0.05)
2000-2003	0.17***	0.21***	-0.01	-0.03**	0.01	0.13	-0.06	0.06
	(0.01)	(0.01)	(0.01)	(0.01)	(0.06)	(0.06)	(0.06)	(0.06)
2004-2007	0.18***	0.23***	0.00	-0.03**	-0.23**	-0.09	-0.06	0.09
	(0.01)	(0.01)	(0.01)	(0.01)	(0.08)	(0.08)	(0.07)	(0.07)
2008-2011	0.16***	0.21***	0.01	-0.03	-0.14	-0.06		
	(0.01)	(0.01)	(0.01)	(0.01)	(0.09)	(0.09)		
2012-2015	0.16***	0.22***	-0.00	-0.04**	0.09	0.25	0.42***	0.65***
	(0.02)	(0.02)	(0.01)	(0.01)	(0.10)	(0.10)	(0.11)	(0.11)
Unemployed	-0.01	-0.10***	0.00	0.10***	-0.50***	-1.42***	-0.13**	-0.67***
(ref: not)[a]	(0.01)	(0.01)	(0.01)	(0.01)	(0.04)	(0.04)	(0.05)	(0.05)
Satisfaction with	0.00	0.00**	-0.00**	-0.00**	0.20***	0.23***	0.19***	0.20***
health[a]	(0.00)	(0.00)	(0.00)	(0.00)	(0.00)	(0.00)	(0.01)	(0.01)
Constant	9.76***	9.75***	0.15***	0.14***	5.12***	4.51***	6.52***	6.23***
	(0.01)	(0.02)	(0.01)	(0.01)	(0.06)	(0.10)	(0.09)	(0.13)
N (individuals)	9,157	8,637	9,157	8,637	9,157	8,637	5,777	5,372

Note: SOEP 1984-2015, v32.1, release 2017. Standard errors in parentheses. *** p<0.001, ** p<0.01, * p<0.05, + p<0.10.
[a] Value of the year before divorce carried forward in all subsequent observations.

Table S2. *Fixed-Effects Models for Housing and Domestic Outcomes*

	Residential Move		Home Ownership		Hours of Housework		Satisfaction with Housework	
	Women	Men	Women	Men	Women	Men	Women	Men
Years before/after divorce (ref.: -3/-5)								
-2/-1	-0.02	-0.03	-0.03***	-0.03	-0.09	-0.01	-0.06	-0.03
	(0.01)	(0.01)	(0.01)	(0.01)	(0.06)	(0.03)	(0.06)	(0.10)
0	0.32***	0.36***	-0.21***	-0.18***	-0.54***	0.34***	0.15	-0.10
	(0.01)	(0.01)	(0.01)	(0.01)	(0.07)	(0.04)	(0.07)	(0.11)
1/2	0.19***	0.14***	-0.25***	-0.22***	-0.66***	0.30***	0.20**	0.04
	(0.01)	(0.01)	(0.01)	(0.01)	(0.06)	(0.03)	(0.06)	(0.10)
3/5	0.11***	0.07***	-0.27***	-0.24***	-0.64***	0.21***	0.12	0.08
	(0.01)	(0.01)	(0.01)	(0.01)	(0.06)	(0.03)	(0.06)	(0.10)
Age (ref.: 21-24)								
25-28	-0.16***	-0.18***	0.09***	0.05***	0.28***	-0.08	-0.32***	-0.07
	(0.01)	(0.02)	(0.01)	(0.01)	(0.06)	(0.05)	(0.06)	(0.15)
29-32	-0.21***	-0.27***	0.20***	0.15***	0.42***	-0.12	-0.47***	-0.19
	(0.01)	(0.02)	(0.01)	(0.01)	(0.06)	(0.06)	(0.06)	(0.15)
33-36	-0.25***	-0.31***	0.33***	0.28***	0.50***	-0.19***	-0.48***	-0.27
	(0.01)	(0.02)	(0.01)	(0.02)	(0.07)	(0.06)	(0.07)	(0.16)
37-40	-0.27***	-0.34***	0.39***	0.38***	0.41***	-0.24***	-0.50***	-0.27
	(0.01)	(0.02)	(0.01)	(0.02)	(0.08)	(0.06)	(0.08)	(0.17)
41-44	-0.28***	-0.36***	0.42***	0.43***	0.22**	-0.25***	-0.50***	-0.26
	(0.01)	(0.02)	(0.01)	(0.02)	(0.09)	(0.06)	(0.09)	(0.18)
45-48	-0.27***	-0.35***	0.42***	0.44***	0.08	-0.27***	-0.44***	-0.20
	(0.02)	(0.02)	(0.01)	(0.02)	(0.10)	(0.07)	(0.10)	(0.20)
49-52	-0.26***	-0.34***	0.41***	0.43***	-0.11	-0.26***	-0.39***	-0.21
	(0.02)	(0.02)	(0.02)	(0.02)	(0.11)	(0.07)	(0.11)	(0.21)
53-56	-0.25***	-0.33***	0.40***	0.42***	-0.25	-0.28***	-0.29	-0.21
	(0.02)	(0.03)	(0.02)	(0.02)	(0.12)	(0.08)	(0.13)	(0.23)
57-60	-0.23***	-0.31***	0.39***	0.41***	-0.38**	-0.18	-0.27	-0.13
	(0.02)	(0.03)	(0.02)	(0.02)	(0.13)	(0.08)	(0.14)	(0.25)
Period (ref.: 1984-1987)								
1988-1991	-0.02***	-0.02***	0.02***	0.02***			-0.19***	-0.13
	(0.01)	(0.01)	(0.00)	(0.00)			(0.03)	(0.07)
1992-1995	-0.03***	-0.03***	0.05***	0.05***	0.07	-0.00	-0.35***	-0.17
	(0.01)	(0.01)	(0.01)	(0.01)	(0.04)	(0.02)	(0.04)	(0.08)
1996-1999	-0.03***	-0.03***	0.08***	0.09***	0.09	0.03	-0.29***	-0.19
	(0.01)	(0.01)	(0.01)	(0.01)	(0.04)	(0.02)	(0.05)	(0.09)
2000-2003	-0.05***	-0.05***	0.12***	0.14***	-0.01	0.07	-0.26***	-0.12
	(0.01)	(0.01)	(0.01)	(0.01)	(0.05)	(0.03)	(0.07)	(0.11)
2004-2007	-0.07***	-0.08***	0.15***	0.17***	-0.03	0.11**	-0.27***	-0.03
	(0.01)	(0.01)	(0.01)	(0.01)	(0.06)	(0.03)	(0.08)	(0.13)
2008-2011	-0.10***	-0.10***	0.17***	0.19***	-0.05	0.14***	-0.22	0.10
	(0.02)	(0.02)	(0.01)	(0.01)	(0.07)	(0.04)	(0.09)	(0.15)
2012-2015	-0.12***	-0.13***	0.20***	0.23***	-0.16	0.15**	-0.24	0.10
	(0.02)	(0.02)	(0.02)	(0.02)	(0.08)	(0.05)	(0.11)	(0.17)
Unemployed	0.02**	-0.01	0.03***	-0.00	0.50***	0.70***	0.02	-0.13
(ref: not)[a]	(0.01)	(0.01)	(0.01)	(0.01)	(0.03)	(0.02)	(0.04)	(0.06)
Satisfaction with	0.00	0.00	0.00	0.00**	-0.00	-0.00	0.21***	0.21***
health[a]	(0.00)	(0.00)	(0.00)	(0.00)	(0.00)	(0.00)	(0.00)	(0.01)
Constant	0.37***	0.46***	0.17***	0.13***	2.96***	0.81***	5.86***	5.65***
	(0.01)	(0.02)	(0.01)	(0.02)	(0.07)	(0.06)	(0.07)	(0.16)
N (individuals)	9,157	8,637	9,157	8,637	8,218	7,682	9,022	7,154

Note: SOEP 1984-2015, v32.1, release 2017. Standard errors in parentheses. *** p<0.001, ** p<0.01, * p<0.05, + p<0.10.
[a] Value of the year before divorce carried forward in all subsequent observations.

Table S3. *Fixed-Effects Models for Health and Well-Being Outcomes*

	Satisfaction with Life		Mental Health		Body Mass Index		Physical Health		Smoking		Drinking	
	Women	Men	Women	Men	Women	Men	Women	Men	Women	Men	Women	Men
Years before/after divorce (ref.: -3/-5)												
-2/-1	-0.39***	-0.28***	-2.20**	-1.23	-0.07	-0.36*	1.28	-0.15	0.01	-0.02	-0.01	0.07
	(0.05)	(0.05)	(0.68)	(0.71)	(0.15)	(0.14)	(0.50)	(0.54)	(0.02)	(0.03)	(0.03)	(0.05)
0	-0.60***	-0.85***	-4.55***	-5.63***	-0.89***	-1.18***	2.99***	2.41***	0.09***	0.02	-0.02	0.08
	(0.06)	(0.06)	(0.87)	(0.87)	(0.19)	(0.18)	(0.65)	(0.67)	(0.03)	(0.03)	(0.04)	(0.06)
1/2	-0.23***	-0.20***	-2.80***	-1.72	-0.38	-0.69***	1.54**	0.46	0.02	0.03	0.01	0.05
	(0.05)	(0.05)	(0.69)	(0.73)	(0.15)	(0.15)	(0.51)	(0.56)	(0.02)	(0.03)	(0.04)	(0.06)
3/5	-0.13**	-0.04	-2.30**	-0.58	-0.20	-0.27	0.61	-0.56	0.00	-0.00	-0.03	0.02
	(0.05)	(0.05)	(0.72)	(0.76)	(0.16)	(0.15)	(0.53)	(0.58)	(0.02)	(0.03)	(0.05)	(0.08)
Age (ref.: 21-24)												
25-28	-0.13**	-0.10	0.81	1.58	0.21	0.71	0.09	-1.46	0.02	-0.04	-0.00	0.10
	(0.05)	(0.07)	(0.97)	(1.37)	(0.22)	(0.28)	(0.72)	(1.05)	(0.03)	(0.05)	(0.05)	(0.11)
29-32	-0.16**	-0.09	0.31	0.14	0.44	1.03***	0.07	-1.42	0.02	-0.09	0.02	0.11
	(0.05)	(0.08)	(1.07)	(1.46)	(0.24)	(0.29)	(0.79)	(1.11)	(0.03)	(0.05)	(0.06)	(0.13)
33-36	-0.16**	-0.14	0.27	-0.00	0.65	1.42***	-0.41	-1.73	0.03	-0.10	0.02	0.09
	(0.06)	(0.08)	(1.15)	(1.53)	(0.26)	(0.31)	(0.85)	(1.17)	(0.03)	(0.05)	(0.07)	(0.14)
37-40	-0.22***	-0.13	0.28	0.39	0.89**	1.64***	-0.94	-1.91	0.03	-0.11	0.04	0.04
	(0.06)	(0.09)	(1.25)	(1.60)	(0.28)	(0.32)	(0.92)	(1.22)	(0.04)	(0.06)	(0.07)	(0.14)
41-44	-0.25***	-0.18	0.40	0.21	1.12***	1.79***	-1.59	-2.02	0.02	-0.11	0.05	0.02
	(0.07)	(0.09)	(1.35)	(1.68)	(0.30)	(0.34)	(1.00)	(1.28)	(0.04)	(0.06)	(0.07)	(0.15)
45-48	-0.31***	-0.22	0.51	0.45	1.38***	2.02***	-2.23	-2.39	0.02	-0.13	0.08	0.03
	(0.08)	(0.10)	(1.47)	(1.76)	(0.33)	(0.35)	(1.09)	(1.35)	(0.04)	(0.06)	(0.08)	(0.15)
49-52	-0.33***	-0.23	0.33	0.35	1.66***	2.28***	-2.86	-2.80	0.02	-0.15	0.08	0.01
	(0.09)	(0.11)	(1.59)	(1.86)	(0.35)	(0.37)	(1.18)	(1.42)	(0.05)	(0.07)	(0.08)	(0.16)
53-56	-0.37***	-0.27	0.89	0.72	1.97***	2.36***	-4.07**	-3.98**	0.01	-0.15	0.09	-0.05
	(0.10)	(0.12)	(1.73)	(1.97)	(0.38)	(0.40)	(1.28)	(1.50)	(0.05)	(0.07)	(0.09)	(0.16)
57-60	-0.35**	-0.19	0.66	1.52	1.98***	2.49***	-5.07***	-4.37**	-0.01	-0.17	0.09	-0.09
	(0.11)	(0.13)	(1.87)	(2.08)	(0.41)	(0.42)	(1.38)	(1.59)	(0.05)	(0.07)	(0.09)	(0.17)
Period (ref.: 1984-1987)												
1988-1991	-0.07**	-0.09***										
	(0.02)	(0.02)										
1992-1995	-0.10**	-0.06										
	(0.03)	(0.03)										
1996-1999	-0.18***	-0.15***										
	(0.04)	(0.04)										
2000-2003	-0.21***	-0.21***										
	(0.05)	(0.05)										
2004-2007	-0.40***	-0.35***	0.67**	0.35	0.26***	0.20***	0.39	0.13	-0.02**	-0.02**		
	(0.06)	(0.06)	(0.23)	(0.22)	(0.05)	(0.04)	(0.17)	(0.17)	(0.01)	(0.01)		
2008-2011	-0.33***	-0.30***	0.76	0.20	0.56***	0.49***	0.32	-0.26	-0.04***	-0.03	-0.00	0.03**
	(0.07)	(0.07)	(0.37)	(0.35)	(0.08)	(0.07)	(0.27)	(0.27)	(0.01)	(0.01)	(0.01)	(0.01)
2012-2015	-0.37***	-0.32***	0.82	-0.18	0.74***	0.62***	-0.07	-0.73	-0.03	-0.06**		
	(0.08)	(0.08)	(0.54)	(0.52)	(0.12)	(0.10)	(0.40)	(0.39)	(0.02)	(0.02)		
Unemployed	-0.21***	-0.87***	-0.19	-2.50***	0.05	0.11	-0.30	0.40	-0.02	0.01	0.00	0.03
(ref: not)[a]	(0.03)	(0.03)	(0.46)	(0.46)	(0.10)	(0.09)	(0.34)	(0.35)	(0.01)	(0.02)	(0.02)	(0.03)
Satisfaction with	0.24***	0.25***	0.93***	0.78***	-0.03**	-0.03**	1.68***	1.67***	0.00	0.00	-0.00	-0.00
health[a]	(0.00)	(0.00)	(0.05)	(0.05)	(0.01)	(0.01)	(0.04)	(0.04)	(0.00)	(0.00)	(0.00)	(0.00)
Constant	6.22***	5.97***	41.95***	44.90***	23.77***	25.02***	41.54***	43.01***	0.26***	0.46***	0.04	0.24
N (individuals)	9,157	8,637	5,756	5,368	5,756	5,368	5,756	5,368	5,756	5,368	2,992	2,708

Note: SOEP 1984-2015, v32.1, release 2017. Standard errors in parentheses. *** p<0.001, ** p<0.01. [a] Value of the year before divorce carried forward in all subsequent observations.

Table S4. *Fixed-Effects Models for Social Outcomes*

	Partner in Household		Single Parenting		Sat. with Family Life		Loneliness		Visiting Relatives		Visiting Friends	
	Women	Men	Women	Men	Women	Men	Women	Men	Women	Men	Women	Men
Years before/after divorce (ref.: -3/-5)												
-2/-1	-0.02***	-0.01	0.01	0.01	-0.80***	-0.53***	0.04	0.06	0.04	-0.00	-0.20**	-0.16
	(0.00)	(0.00)	(0.00)	(0.00)	(0.11)	(0.12)	(0.04)	(0.04)	(0.06)	(0.06)	(0.06)	(0.06)
0	-0.80***	-0.79***	0.54***	0.13***	-1.66***	-2.44***	0.05	0.32***	-0.04	0.06	0.03	0.19**
	(0.00)	(0.00)	(0.00)	(0.00)	(0.12)	(0.13)	(0.05)	(0.05)	(0.06)	(0.07)	(0.06)	(0.07)
+1/+2	-0.74***	-0.69***	0.52***	0.13***	-0.66***	-1.00***	0.03	0.13**	-0.00	0.01	-0.02	0.12
	(0.00)	(0.00)	(0.00)	(0.00)	(0.11)	(0.12)	(0.05)	(0.04)	(0.05)	(0.05)	(0.05)	(0.05)
+3/+5	-0.61***	-0.59***	0.44***	0.10***	-0.47***	-0.30	-0.05	0.16***	0.00	0.01	-0.12	0.04
	(0.00)	(0.00)	(0.00)	(0.00)	(0.12)	(0.13)	(0.05)	(0.05)	(0.05)	(0.06)	(0.05)	(0.06)
Age (ref.: 21-24)												
25-28	0.01	0.03***	-0.00	-0.02***	-0.23	-0.19	0.09*	0.04	0.04	0.00	-0.04	-0.09
	(0.00)	(0.01)	(0.00)	(0.00)	(0.13)	(0.22)	(0.03)	(0.05)	(0.05)	(0.08)	(0.05)	(0.08)
29-32	0.02***	0.04***	-0.02***	-0.02***	-0.34	-0.35	0.07	0.03	0.07	-0.02	-0.02	-0.16
	(0.00)	(0.01)	(0.00)	(0.00)	(0.15)	(0.24)	(0.04)	(0.06)	(0.05)	(0.08)	(0.05)	(0.08)
33-36	0.03***	0.05***	-0.02***	-0.03***	-0.39	-0.57	0.06	0.04	0.04	-0.06	0.00	-0.19
	(0.00)	(0.01)	(0.00)	(0.00)	(0.16)	(0.24)	(0.05)	(0.06)	(0.06)	(0.09)	(0.07)	(0.09)
37-40	0.02***	0.05***	-0.02***	-0.03***	-0.53**	-0.76**	0.05	0.04	-0.00	-0.05	-0.04	-0.20
	(0.00)	(0.01)	(0.00)	(0.00)	(0.17)	(0.25)	(0.05)	(0.06)	(0.08)	(0.10)	(0.08)	(0.10)
41-44	0.02***	0.05***	-0.01*	-0.03***	-0.70***	-0.86***	0.04	0.03	0.00	-0.02	-0.05	-0.21
	(0.01)	(0.01)	(0.01)	(0.00)	(0.17)	(0.26)	(0.06)	(0.07)	(0.09)	(0.12)	(0.10)	(0.11)
45-48	0.02***	0.05***	-0.01	-0.02***	-0.78***	-0.93***	0.05	0.01	0.03	0.00	-0.04	-0.23
	(0.01)	(0.01)	(0.01)	(0.00)	(0.18)	(0.26)	(0.07)	(0.08)	(0.11)	(0.13)	(0.11)	(0.13)
49-52	0.02***	0.04***	-0.01	-0.02***	-0.76***	-1.06***	0.03	0.03	0.06	0.08	-0.05	-0.22
	(0.01)	(0.01)	(0.01)	(0.00)	(0.20)	(0.27)	(0.08)	(0.08)	(0.13)	(0.15)	(0.13)	(0.14)
53-56	0.02**	0.04***	-0.01	-0.02***	-0.78***	-1.03***	0.02	0.03	0.15	0.16	-0.06	-0.23
	(0.01)	(0.01)	(0.01)	(0.01)	(0.21)	(0.28)	(0.09)	(0.09)	(0.14)	(0.16)	(0.15)	(0.16)
57-60	0.02**	0.04***	-0.02	-0.02***	-0.71**	-1.10***	-0.01	-0.00	0.17	0.25	-0.01	-0.18
	(0.01)	(0.01)	(0.01)	(0.01)	(0.22)	(0.29)	(0.09)	(0.10)	(0.16)	(0.18)	(0.16)	(0.18)
Period (ref.: 1984-1987)												
1988-1991	-0.00	0.00	-0.00	0.00								
	(0.00)	(0.00)	(0.00)	(0.00)								
1992-1995	0.00	0.01**	-0.00	-0.00					-0.01	-0.05	0.03	0.00
	(0.00)	(0.00)	(0.00)	(0.00)					(0.03)	(0.03)	(0.03)	(0.03)
1996-1999	0.00	0.00	-0.00	-0.00			-0.00	-0.01	-0.03	-0.10	0.00	0.02
	(0.00)	(0.00)	(0.00)	(0.00)			(0.01)	(0.01)	(0.04)	(0.04)	(0.04)	(0.04)
2000-2003	0.00	0.01	-0.00	-0.00					-0.06	-0.17**	-0.02	-0.01
	(0.00)	(0.00)	(0.00)	(0.00)					(0.06)	(0.06)	(0.06)	(0.06)
2004-2007	-0.00	0.00	-0.00	-0.00								
	(0.00)	(0.00)	(0.00)	(0.00)								
2008-2011	-0.00	0.00	-0.00	-0.00	0.11***	0.10***	0.04	0.04	-0.11	-0.23**	-0.08	-0.08
	(0.01)	(0.01)	(0.01)	(0.00)	(0.03)	(0.03)	(0.04)	(0.04)	(0.08)	(0.09)	(0.08)	(0.09)
2012-2015	-0.00	0.00	-0.00	-0.00	0.01	0.03	0.03	0.02	-0.19	-0.34**	-0.16	-0.13
	(0.01)	(0.01)	(0.01)	(0.00)	(0.04)	(0.04)	(0.05)	(0.05)	(0.10)	(0.11)	(0.10)	(0.11)
Unemployed	-0.00	0.00	0.00	0.00	0.11	0.02	0.04	0.08***	0.05	0.05	0.04	-0.00
(ref: not)[a]	(0.00)	(0.00)	(0.00)	(0.00)	(0.06)	(0.06)	(0.02)	(0.02)	(0.03)	(0.03)	(0.03)	(0.03)
Satisfaction with	-0.00	-0.00	-0.00	-0.00	0.16***	0.15***	-0.02***	-0.01***	0.00	0.01	0.01	-0.00
health[a]	(0.00)	(0.00)	(0.00)	(0.00)	(0.01)	(0.01)	(0.00)	(0.00)	(0.00)	(0.00)	(0.00)	(0.00)
Constant	0.98***	0.95***	0.02***	0.03***	7.61***	7.94***	0.22***	0.13	0.54***	0.52***	0.50***	0.61***
N (individuals)	9,157	8,637	9,157	8,637	6,017	5,613	4,396	4,013	6,578	6,034	6,580	6,036

Note: SOEP 1984-2015, v32.1, release 2017. Standard errors in parentheses. *** p<0.001, ** p<0.01. [a] Value of the year before divorce carried forward in all subsequent observations.

Table S5. *Fully Interacted Fixed-Effects Models for Economic Outcomes and Housing/Domestic Outcomes*

	Annual Household Income (log)	Risk of Poverty	Satisfaction with Income	Satisfaction with Standard of Living	Residential Move	Home Ownership	Hours of Housework	Satisfaction with Housework
Years before/after divorce								
-2/-1	0.01	0.01	0.02	-0.10	-0.03	-0.03	-0.01	-0.03
	(0.01)	(0.01)	(0.07)	(0.08)	(0.01)	(0.01)	(0.05)	(0.09)
0	0.07***	-0.01	-0.41***	-0.70***	0.36***	-0.18***	0.34***	-0.10
	(0.01)	(0.01)	(0.08)	(0.09)	(0.01)	(0.01)	(0.06)	(0.10)
+1/+2	0.06***	-0.01	-0.36***	-0.54***	0.14***	-0.22***	0.30***	0.04
	(0.01)	(0.01)	(0.07)	(0.08)	(0.01)	(0.01)	(0.05)	(0.09)
	0.04***	0.01	-0.24***	-0.31***	0.07***	-0.24***	0.21***	0.08
	(0.01)	(0.01)	(0.07)	(0.08)	(0.01)	(0.01)	(0.05)	(0.09)
Years before/after divorce x Female								
-2/-1	-0.02	-0.00	-0.07	0.06	0.01	-0.01	-0.08	-0.02
	(0.01)	(0.01)	(0.09)	(0.10)	(0.02)	(0.01)	(0.07)	(0.11)
0	-0.61***	0.38***	-0.71***	0.01	-0.04	-0.03	-0.88***	0.25
	(0.02)	(0.01)	(0.11)	(0.13)	(0.02)	(0.02)	(0.08)	(0.13)
+1/+2	-0.43***	0.27***	-0.36***	-0.06	0.05**	-0.03	-0.95***	0.16
	(0.01)	(0.01)	(0.09)	(0.11)	(0.02)	(0.01)	(0.07)	(0.11)
+3/+5	-0.32***	0.18***	-0.26**	-0.05	0.04	-0.03	-0.85***	0.05
	(0.01)	(0.01)	(0.09)	(0.11)	(0.02)	(0.01)	(0.07)	(0.11)

Note: SOEP 1984-2015, v32.1, release 2017. Standard errors in parentheses. *** $p<0.001$, ** $p<0.01$. Models control for all covariates shown in tables A1-A4 and interactions between these covariates and gender.

Table S6. *Fully Interacted Fixed-Effects Models for Health/Well-Being Outcomes and Social Outcomes*

	Satisfaction with Life	Mental Health	Body Mass Index	Physical Health	Smoking	Drinking	Partner in Household	Single Parenting	Sat. with Family Life	Loneli-ness	Visiting Relatives	Visiting Friends
Years before/after divorce												
-2/-1	-0.28***	-1.23	-0.36	-0.15	-0.02	0.07	-0.01	0.01	-0.53***	0.06	-0.00	-0.16
	(0.05)	(0.74)	(0.16)	(0.56)	(0.02)	(0.04)	(0.00)	(0.00)	(0.12)	(0.04)	(0.06)	(0.06)
0	-0.85***	-5.63***	-1.18***	2.41***	0.02	0.08	-0.79***	0.13***	-2.44***	0.32***	0.06	0.19**
	(0.06)	(0.91)	(0.19)	(0.68)	(0.03)	(0.06)	(0.00)	(0.00)	(0.13)	(0.05)	(0.07)	(0.07)
+1/+2	-0.20***	-1.72	-0.69***	0.46	0.03	0.05	-0.69***	0.13***	-1.00***	0.13**	0.01	0.12
	(0.06)	(0.77)	(0.16)	(0.57)	(0.02)	(0.05)	(0.00)	(0.00)	(0.12)	(0.05)	(0.05)	(0.05)
+3/+5	-0.04	-0.58	-0.27	-0.56	-0.00	0.02	-0.59***	0.10***	-0.30	0.16**	0.01	0.04
	(0.05)	(0.79)	(0.17)	(0.59)	(0.03)	(0.06)	(0.00)	(0.00)	(0.13)	(0.05)	(0.06)	(0.06)
Years before/after divorce x Female												
-2/-1	-0.10	-0.97	0.29	1.43	0.03	-0.08	-0.01	0.00	-0.28	-0.02	0.05	-0.04
	(0.07)	(0.99)	(0.21)	(0.74)	(0.03)	(0.06)	(0.01)	(0.00)	(0.16)	(0.06)	(0.09)	(0.09)
0	0.25**	1.08	0.29	0.58	0.06	-0.10	-0.01	0.41***	0.77***	-0.27***	-0.10	-0.15
	(0.09)	(1.24)	(0.26)	(0.93)	(0.04)	(0.08)	(0.01)	(0.01)	(0.17)	(0.07)	(0.09)	(0.09)
+1/+2	-0.03	-1.08	0.31	1.08	-0.01	-0.04	-0.05***	0.38***	0.34	-0.10	-0.01	-0.14
	(0.07)	(1.02)	(0.22)	(0.76)	(0.03)	(0.07)	(0.01)	(0.00)	(0.16)	(0.06)	(0.07)	(0.07)
+3/+5	-0.09	-1.72	0.07	1.18	0.01	-0.05	-0.02***	0.34***	-0.17	-0.22**	-0.00	-0.16
	(0.07)	(1.05)	(0.22)	(0.79)	(0.03)	(0.09)	(0.01)	(0.00)	(0.18)	(0.07)	(0.08)	(0.08)

Note: SOEP 1984-2015, v32.1, release 2017. Standard errors in parentheses. *** p<0.001, ** p<0.01. Models control for all covariates shown in tables A1-A4 and interactions between these covariates and gender.

Table S7. *F-Tests for Change in R-Squared*

Economic outcomes	F	prob > F
Satisfaction with standard of living	0.39	0.8168
Satisfaction with income	13.66	0.0000
Annual household income	532.75	0.0000
Poverty	301.21	0.0000
Housing and domestic outcomes		
Residential moves	7.26	0.0000
Home ownership	2.57	0.0357
Satisfaction with housework	1.75	0.1350
Hours of housework	82.35	0.0000
Health and well-being outcomes		
Satisfaction with life	5.24	0.0003
Mental health	1.68	0.1523
Physical health	1.07	0.3699
BMI	0.95	0.4317
Smoking	1.32	0.2589
Drinking	0.83	0.5066
Social outcomes		
Partner	21.19	0.0000
Single parenting	3661.70	0.0000
Satisfaction with family life	17.42	0.0000
Loneliness	6.37	0.0000
Visiting relatives	0.53	0.7170
Visiting friends	2.30	0.0559

Note: Tests based on models estimated jointly for men and women, including the covariates shown in Tables A1-A4. Reference models are fully interacted by gender, except for the interactions between the four dummy variables for "Years before/after divorce" and gender. Model fit comparisons are shown between the reference models and models for which the interaction between "Years before/after divorce" and gender are added.
